# Supplementary material for: Usefulness of ß-d-Glucan Assay for the First-Line Diagnosis of Pneumocystis Pneumonia and for Discriminating between Pneumocystis Colonization and Pneumocystis Pneumonia
Source: J Fungi (Basel). 2022 Jun 24;8(7):663. doi: 10.3390/jof8070663 (PMC9318034; doi:10.3390/jof8070663)
Supplement: Supplementary file 1 [file jof-08-00663-s001.zip › jof-1744729-supplementary.pdf]

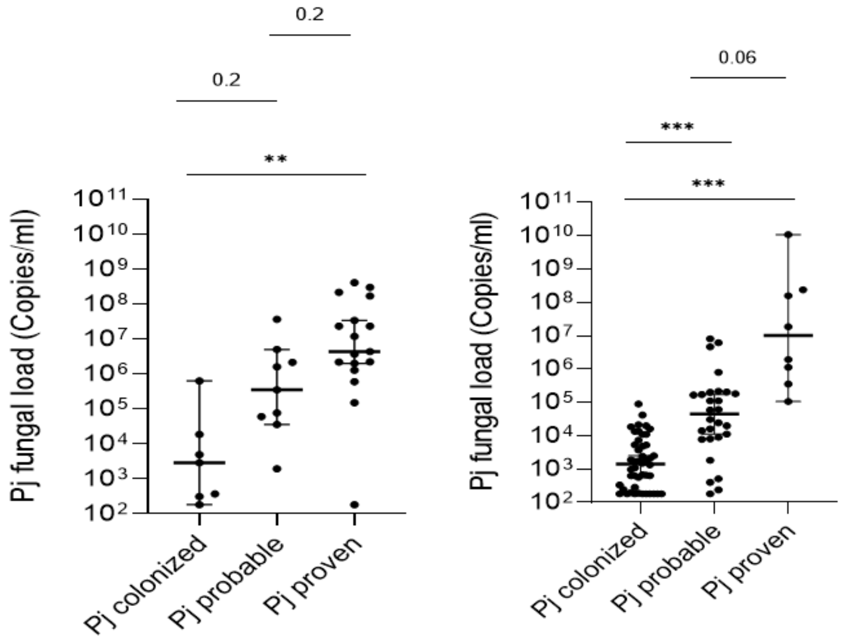

Figure S1. Comparison of Pj fungal load (median and CI 95) in episodes of proven PjP, probable PjP and Pj colonization according to HIV-status of the patient. (a) episodes occuring in HIV-positive patients and (b): in HIV-negative patients. Comparison of the three groups was analyzed using Kruskal-Wallis test followed by Dunn's multiple comparison test. * < 0.001, ** < 0.0001.
